# Supplementary material for: A Contact-Free Optical Device for the Detection of Pulmonary Congestion—A Pilot Study
Source: Biosensors (Basel). 2022 Oct 6;12(10):833. doi: 10.3390/bios12100833 (PMC9599847; doi:10.3390/bios12100833)
Supplement: Supplementary file 1 [file biosensors-12-00833-s001.zip › biosensors-1858465-supplementary.pdf]

Supplementary Data:

Table S1: Baseline Characteristics (Subgroup Analysis)

Characteristics	Total Population [n=170]	Lung Congestion	
		No [n=126]	Yes [n=44]
Age [years]: mean (SD)	67(14)	65(15)	74(10)
Gender (males): n (%)	109(64%)	76(60%)	33(75%)
Gender (female): n (%)	61(36%)	50(40%)	11(25%)
Height [cm]: mean (SD)	167(9)	167(10)	167(9)
Weight [Kg]: mean (SD)	79(15)	79(16)	79(13)
BMI [Kg/m^2]: mean (SD)	28(5)	28(5)	29(5)
BMI ⩾ 30: n (%)	50(29%)	34(27%)	16(36%)
Smoking: n (%)	25(15%)	18(14%)	7(16%)
**Risk Factors:**			
Heart failure: n (%)	112(66%)	76(60%)	36(82%)
Hypertension: n (%)	90(53%)	53(42%)	37(84%)
Diabetes: n (%)	46(27%)	22(17%)	24(55%)
Stroke / TIA / thromboembolism: n (%)	12(7%)	7(6%)	5(11%)
Vascular disease: n (%)	85(50%)	56(44%)	29(66%)
**Other Medical Conditions:**			
Respiratory conditions (COPD, asthma, pulmonary HTN, pulmonary embolism, lung cancer etc).	26(15%)	11(9%)	15(34%)
Valvular disorders (any type; mild, moderate or severe)	61(36%)	29(23%)	32(73%)
**Medications:**			
Anticoagulant: n (%)	39(23%)	23(18%)	16(36%)
Antiplatelet: n (%)	101(59%)	73(58%)	28(64%)
ACEi: n (%)	43(25%)	35(28%)	8(18%)
ARB: n (%)	19(11%)	11(9%)	8(18%)
CCB: n (%)	30(18%)	14(11%)	16(36%)
Beta-blocker: n (%)	92(54%)	65(52%)	27(61%)
Diuretic (any): n (%)	68(40%)	36(29%)	32(73%)
Antiarrhythmic: n (%)	15(9%)	6(5%)	9(20%)

BMI: body mass index; TIA: transient ischemic attack; COPD: chronic obstructive pulmonary disease; HTN: hypertension; ACEi: angiotensin-converting enzyme inhibitor; ARB: angiotensin receptor blocker; CCB: calcium channel blocker.

Table S2: Diagnostic accuracy of the Gili lung congestion algorithm, when including measurements with inconclusive predictions.

Variable	Original Point Estimates [95% CI]	Point Estimates When Including Inconclusive Predictions (Worse-Case Scenario)

			[95% CI]	
	**Complete Study Population**	**Subgroup Analysis**	**Complete Study Population**	**Subgroup Analysis**
Sensitivity	0.91 [0.86, 0.93]	0.99 [0.96, 1]	0.91 [0.87, 0.94]	0.99 [0.96, 1]
Specificity	0.91 [0.87, 0.94]	0.93 [0.88, 0.95]	0.88 [0.84, 0.91]	0.89 [0.84, 0.93]
PPV_{Sample}	0.92 [0.88, 0.95]	0.89 [0.83, 0.93]	0.90 [0.86, 0.93]	0.84 [0.79, 0.89]
NPV_{Sample}	0.90 [0.85, 0.93]	0.99 [0.97, 1]	0.90 [0.85, 0.93]	0.99 [0.97, 1]
$PPV_{32\%}$	0.82 [0.77, 0.86]	0.86 [0.80, 0.90]	0.78 [0.73, 0.83]	0.81 [0.75, 0.86]
$NPV_{32\%}$	0.95 [0.92, 0.97]	0.99 [0.97, 1]	0.95 [0.92, 0.97]	0.99 [0.97, 1]
PLR	10 [6.6, 15]	9.9 [6.4, 15]	7.7 [5.4, 11]	8.9 [5.9, 13]
NLR	0.1 [0.07, 0.16]	0.015 [0.005, 0.049]	0.1 [0.066, 0.15]	0.015 [0.005, 0.048]

CI: Confidence Intervals

PPV: Positive predictive value

NPV: Negative predictive value

Sample: Based on prevalence from study sample set

32%: Based on expected prevalence of 32% [24]

PLR: Positive likelihood ratio

NLR: Negative likelihood ratio
